# Differentiation Between Suicide Attempt and Suicidal Ideation in Patients With Major Depressive Disorder Using Cortical Functional Network

**DOI:** 10.1192/bjo.2024.702

**Published:** 2024-08-01

## Abstract

Differentiation between suicide attempt and suicidal ideation in patients with major depressive disorder using cortical functional network Sehoon Shim, Differentiation between suicide attempt and suicidal ideation in patients with major depressive disorder using cortical functional network Youngjoon Kwon.

Department of Psychiatry, Soonchunhyang University Cheonan Hospital, Soonchunhyang University College of Medicine, Cheonan, Korea, Republic of Korea.

**Aims:**

Studies exploring the neurophysiology of suicide are scarce, and the neuropathology of related suicide is poorly understood. This study investigated source-level cortical functional networks using resting-state electroencephalography (EEG) in drug-naive patients with suicide attempt and suicide ideation.

**Methods:**

EEG was recorded in 55 patients with suicide attempt and 54 patients with suicide ideation. Graph theory-based source-level weighted functional networks were assessed via strength, clustering coefficient (CC), and path length (PL) in seven frequency bands. This study applied machine learning to differentiate the two groups using source-level network features.

**Results:**

At the global level, patients with suicide attempt showed lower strength and CC, and higher PL in the high alpha band, compared to those with suicide ideation. At the nodal level, compared to suicide ideation, patients with suicide attempt showed lower high alpha band nodal CCs in most of brain regions. The best classification performance for suicide attempt and suicide ideation showed an accuracy of 73.39%, a sensitivity of 76.36%, and a specificity of 70.37% based on high alpha band network features.

**Conclusion:**

Our findings suggest that abnormal high alpha band functional network reflects the pathophysiological characteristics of suicide and might serve clinically as a neuromarker of suicide.

